# The ATHENA Competency Framework: An Evaluation of Its Validity According to Instructional Designers and Human Resource Development Professionals

**DOI:** 10.3390/jintelligence14020023

**Published:** 2026-02-03

**Authors:** Jeremy Lamri, Karin Valentini, Felipe Zamana, Todd Lubart

**Affiliations:** 1 Tomorrow Theory, 75002 Paris, France; 2 The Aix-Marseille Graduate School of Management, Aix-Marseille University, 13100 Aix-en-Provence, France; 3 Laboratoire de Psychologie et d’Ergonomie Appliquées (LaPEA), Université Paris Cité and Univ Gustave Eiffel, 92100 Boulogne-Billancourt, France

**Keywords:** ATHENA framework, competency framework, instructional design, HR development, facet clarity, multidimensional skills, framework validation

## Abstract

The ATHENA (Advanced Tool for Holistic Evaluation and Nurturing of Abilities) competency framework proposes a multidimensional approach to human performance structured around five interdependent dimensions (cognition, conation, knowledge, emotion, and sensori-motion), operationalized through 60 fine-grained facets. Although ATHENA is grounded in contemporary psychological theory and supported conceptually by multivariate research in intelligence, creativity, and skill acquisition, empirical evidence regarding the clarity and practical comprehensibility of its facets remains limited. This study investigates the extent to which instructional designers and human resource development (HRD) professionals—two groups who routinely operationalize competencies for learning, assessment, and workforce development—understand and evaluate the semantic clarity and usability of the 60 facets. Seventy-five practitioners completed a structured evaluation of the ATHENA framework facets, which are designed to be used in a hybrid intelligence system for competency management. This article presents the theoretical background, methodological design, and results concerning users’ comprehension of the framework’s components. The findings support, in general, the compatibility of ATHENA’s facets and practitioners’ conceptions.

## 1. Introduction

Competency frameworks are widely used to support learning design, assessment, and talent development, yet they frequently rely on heterogeneous and partially incompatible construct traditions. In practice, competencies are often decomposed into categories such as knowledge, skills, and attitudes, or “hard” versus “soft” skills, even though these distinctions remain theoretically contested and empirically porous (Cimatti, 2016; Tardif, 2006; Lamri & Lubart, 2023). This conceptual fragmentation complicates both scientific accumulation and applied implementation: overlapping labels, unstable definitions, and unclear construct boundaries can weaken communication, assessment coherence, and downstream instructional decisions (Eraut, 2004; Sandberg, 2000; Green, 2024).

Recent approaches using intelligence, creativity, and skill acquisition increasingly emphasize that high-level performance emerges from coordinated resources that span cognitive processes, motivation and volition, emotion regulation, knowledge structures, and embodied capacities (Deci & Ryan, 2000; Gross, 2002; Varela et al., 1991). However, many large-scale taxonomies and occupational frameworks—while highly useful for standardization—remain primarily descriptive and do not necessarily provide a psychologically integrated account of how internal resources combine to produce competence (e.g., *ONET*; *ESCO*; Fleishman & Reilly, 1992; National Center for O*NET Development, 2023; European Commission, 2024). As a result, practitioners may rely on “folk” interpretations when translating competency labels into learning objectives or development plans, with direct consequences for the quality and validity of applied decisions (Shavelson & Huang, 2003; Billett, 2020).

The ATHENA (Advanced Tool for Holistic Evaluation and Nurturing of Abilities) competency framework was developed to offer an integrative representation of competence, structured around five interdependent dimensions—cognition, conation, knowledge, emotion, and sensori-motion—operationalized through 60 facets (Lamri et al., 2025). All 60 facets are described in Appendix A. ATHENA’s theoretical claim is not that competencies are discrete traits, but that they reflect context-sensitive configurations of internal resources that support adaptive performance. Such integrative architectures, however, face a recurrent challenge: before any psychometric modeling or predictive validation can be meaningfully undertaken, the framework’s basic conceptual units must be interpretable, distinguishable, and usable by those who operationalize them in real settings.

Accordingly, the present paper focuses on semantic clarity and conceptual distinctiveness at the facet level. In this context, *semantic clarity* refers to whether a facet label and its definition are understood as intended and perceived as being sufficiently precise for applied use. *Conceptual distinctiveness* refers to whether practitioners can differentiate facets and locate them within the framework’s intended dimensional architecture, rather than collapsing them into broader or more familiar categories. These properties correspond to early forms of content- and face-validity evidence: they do not establish factorial structure, reliability, or predictive validity, but they constitute a necessary prerequisite for subsequent validation steps (Haynes et al., 1995; Nevo, 1985).

To examine this prerequisite, we conducted an expert-based evaluation of all 60 ATHENA facets with two practitioner populations who routinely translate competency constructs into operational decisions: instructional designers and human resource development (HRD) professionals. Participants rated each facet definition on (a) perceived appropriateness and (b) alignment with their expectations, and they assigned each facet to one of ATHENA’s five dimensions. This design allows us to identify (i) whether the facets are generally interpretable and acceptable to expert users, and (ii) where systematic ambiguities or boundary issues emerge, particularly in dimensional placement.

Three research questions guided the study:

- RQ1: To what extent do practitioners perceive the ATHENA facets as semantically clear and understandable, as reflected in judgments of definition appropriateness?
- RQ2: To what extent do the facet definitions align with practitioners’ expectations, indicating conceptual familiarity or divergence?
- RQ3: Are certain dimensions of the ATHENA framework more difficult for practitioners to interpret, as reflected in systematic patterns of facet misclassification across dimensions? These questions are intentionally descriptive and exploratory. They are designed to capture patterns of agreement, divergence, and conceptual tension, rather than to test confirmatory hypotheses about the framework’s structure.

Importantly, our aim is not to claim psychometric validation of ATHENA. Instead, we provide an exploratory assessment of practitioner-based interpretability and conceptual boundary signals, which can inform the refinement of facet labels/definitions and support a coherent roadmap toward later psychometric and performance-based validation.

## 2. Background and Theoretical Rationale

### 2.1. Conceptual Fragmentation in Competency Frameworks

Research in psychology and education has long established that competent performance emerges from the interaction of multiple internal resources, rather than from isolated traits or skills. Cognitive processes, motivational dynamics, emotional regulation, knowledge structures, and embodied capacities jointly contribute to adaptive behavior across domains (Deci & Ryan, 2000; Gross, 2002; Varela et al., 1991). Despite this consensus, many applied competency frameworks continue to rely on simplified categorical distinctions—such as knowledge, skills, and attitudes (KSAOs), or hard versus soft skills—that remain theoretically unstable and empirically overlapping (Cimatti, 2016; Tardif, 2006).

Large-scale occupational taxonomies such as *ONET* and *ESCO* provide valuable standardized descriptors of work activities, skills, and qualifications, enabling comparability across jobs and labor markets (Fleishman & Reilly, 1992; National Center for O*NET Development, 2023; European Commission, 2024). However, their primary function is classificatory, rather than explanatory. These systems describe *what* skills are associated with occupations but do not aim to model *how* internal psychological resources combine to produce competent performance, nor how these resources can be systematically developed through learning or training.

A similar limitation characterizes many soft-skills frameworks. Systematic reviews highlight extensive conceptual inflation, with dozens of overlapping labels used to describe partially redundant constructs. In applied contexts, this inflation often translates into vague competency statements that are difficult to operationalize, assess, or develop. For practitioners, such ambiguity increases their reliance on tacit interpretations and professional intuition, which can undermine consistency and transparency in instructional design and talent development (Eraut, 2004; Sandberg, 2000).

These limitations point to a central challenge: integrative competency frameworks must not only be theoretically grounded but also conceptually explicit at a level that allows practitioners to distinguish, interpret, and operationalize competency components in a consistent manner.

### 2.2. The ATHENA Framework: Integrative Architecture and Granularity

The ATHENA framework was developed to address these challenges by proposing a multidimensional and agentic conception of competence (Pessoa, 2022; Lamri et al., 2025). ATHENA conceptualizes competence as an emergent pattern of performance, resulting from the coordinated activation of internal resources across five dimensions: cognition, conation, knowledge, emotion, and sensori-motion. Rather than treating competencies as static entities, the framework emphasizes their context-sensitive and developmental nature.

ATHENA aligns with multivariate approaches in intelligence and creativity research, which highlight the joint contribution of cognitive, motivational, emotional, and environmental factors in high-level performance (Lubart et al., 2015). Its distinctive contribution lies not in introducing new dimensions per se, but in offering a systematic decomposition of these dimensions into 60 fine-grained facets that are designed for applied use.

The rationale for this level of granularity is twofold. First, instructional design and talent development require constructs that are sufficiently precise to inform concrete decisions, such as selecting pedagogical strategies, designing learning pathways, or identifying developmental priorities (Pellegrino & Hilton, 2012; Mulder, 2014). Broad dimensions alone are often too coarse to support such decisions. Second, finer-grained facets make explicit the internal resources that are typically implicit or conflated in higher-level competency labels, thereby reducing semantic ambiguity and facilitating more transparent communication between theory and practice.

Importantly, ATHENA does not claim that the 60 facets represent an exhaustive or definitive taxonomy of human competence. Rather, they constitute a structured hypothesis: a proposed set of conceptual building blocks that aim to balance theoretical coherence with practical usability. As such, the framework’s validity depends not only on its theoretical grounding but also on whether these facets are interpretable and distinguishable for expert users.

The facet definitions were developed through an iterative conceptual synthesis, rather than through inductive item generation or empirical scale construction. The process involved three main criteria.

First, each facet corresponds to a recurrent functional resource identified in established psychological studies (e.g., reasoning, motivation, emotion regulation, procedural knowledge, sensorimotor coordination), rather than to context-specific behaviors or occupational descriptors.

Second, definitions were formulated to emphasize the functional role of each resource in adaptive performance, focusing on what the resource enables an individual to do across contexts, rather than on its measurement properties.

Third, particular attention was paid to applied interpretability: definitions were intentionally concise, context-sensitive, and designed to be usable by practitioners in instructional and developmental settings, even at the cost of theoretical exhaustiveness.

Accordingly, the definitions should be understood as theoretically grounded operational hypotheses, whose adequacy and distinctiveness are precisely what the present study seeks to examine.

### 2.3. Why Semantic Interpretability Constitutes a Necessary Validation Step

Before engaging in psychometric modeling, factor analysis, or predictive validation, it is necessary to establish whether the basic units of a framework are semantically clear and conceptually interpretable. Several authors emphasize the necessity of establishing content-level clarity and construct interpretability (Haynes et al., 1995; Boateng et al., 2018; Almanasreh et al., 2019). In complex, multidimensional frameworks, insufficiently specified conceptual units may lead to artificial factor structures or misleading quantitative results, as measurement models risk capturing semantic noise, rather than psychologically meaningful constructs.

This issue is particularly salient for multidimensional frameworks that explicitly aim to go beyond cognition-centric models. Practitioners’ tendencies to reassign facets toward familiar domains (e.g., cognition) may reveal either genuine conceptual overlap or insufficiently explicit theoretical distinctions. In both cases, such patterns are informative. They identify zones where conceptual boundaries require clarification, refinement, or empirical re-examination before stronger validation claims can be made (Borsboom et al., 2004).

From a conceptual standpoint, this concern echoes broader discussions on construct explication and theoretical clarity in psychology (Cronbach & Meehl, 1955; Slaney & Racine, 2017). These authors argue that constructs must be sufficiently explicit, differentiated, and communicable before they can be meaningfully operationalized or measured.

Accordingly, the present study focuses on practitioner-based semantic evaluation as a preliminary validation step. By examining how instructional designers and HRD professionals interpret, differentiate, and categorize ATHENA’s facets, the study aims to identify both areas of conceptual robustness and points of friction that warrant further theoretical or empirical work.

## 3. Contextual Note on the Broader ATHENA System

The ATHENA competency framework was originally developed as the conceptual foundation of a broader applied system that was designed to support instructional design and talent development in organizational contexts. This system includes several modules that are intended to assist practitioners in task analysis, cohort characterization, and learning pathway design. These components rely on the ATHENA framework as an underlying conceptual ontology.

However, it is important to clarify that the present study does not evaluate the ATHENA system itself, nor its AI-supported functionalities. No claims are made here regarding system performance, instructional effectiveness, or algorithmic validity. The system is mentioned solely to situate the framework within its applied context and to explain why practitioner-based interpretability is a critical prerequisite for downstream applications.

From a methodological standpoint, the current investigation focuses exclusively on the semantic clarity, conceptual distinctiveness, and dimensional interpretability of the framework’s 60 facets. These properties are necessary for any subsequent use of the framework in applied or computational settings, but they are not sufficient to establish the validity of the system as a whole. Any evaluation of AI-assisted processes, automated recommendations, or learning outcomes would require dedicated empirical studies with appropriate behavioral and performance-based measures.

Accordingly, references to the broader ATHENA system should be understood as contextual, rather than evidential. The present article is intentionally limited to examining whether the conceptual units that constitute the framework are interpretable and distinguishable for expert users, prior to any psychometric or system-level validation.

## 4. The Present Study

The aim of the present study is to provide an exploratory, expert-based assessment of the semantic clarity, conceptual distinctiveness, and dimensional interpretability of the 60 facets constituting the ATHENA competency framework. This investigation is explicitly situated as a preliminary validation step, focusing on content-level interpretability rather than on psychometric structure, reliability, or predictive validity.

In the development of complex, multidimensional competency frameworks, early validation efforts are often limited to internal theoretical coherence or immediately oriented toward quantitative modeling. However, without prior evidence that the proposed conceptual units are interpretable and distinguishable for expert users, subsequent psychometric analyses risk operating on constructs that are semantically unstable or inconsistently understood. The present study addresses this gap by examining how professionals who routinely operationalize competencies interpret ATHENA’s facets when confronted with their definitions and dimensional structure.

### 4.1. Rationale for an Expert-Based Semantic Evaluation

Instructional designers and human resource development (HRD) professionals occupy a pivotal position between psychological theory and applied decision-making. They are responsible for translating abstract competency constructs into learning objectives, assessment criteria, developmental pathways, and organizational tools. Their judgments therefore provide a relevant perspective for assessing whether a competency framework’s conceptual units are sufficiently clear and usable for applied contexts.

From a validity standpoint, expert evaluations of definition clarity, expectation alignment, and categorical placement contribute evidence related to content validity and face validity (Haynes et al., 1995; Nevo, 1985; Boateng et al., 2018). Although such evidence is necessarily limited and subjective, it constitutes a necessary condition for further validation stages. If experts cannot reliably interpret facet definitions or agree on their conceptual location within a framework, subsequent efforts to measure, model, or predict competence using these facets would be undermined.

Accordingly, the present study does not attempt to establish the internal factorial structure of the ATHENA framework, nor does it test behavioral or performance-based outcomes. Instead, it seeks to identify whether the framework’s basic conceptual units function as interpretable building blocks from the perspective of expert users, and where systematic ambiguities or boundary issues emerge.

### 4.2. Research Questions

Three research questions guided the study:

- RQ1: To what extent do practitioners perceive the ATHENA facets as semantically clear and understandable, as reflected in judgments of definition appropriateness?
- RQ2: To what extent do the facet definitions align with practitioners’ expectations, indicating conceptual familiarity or divergence?
- RQ3: Are certain dimensions of the ATHENA framework more difficult for practitioners to interpret, as reflected in systematic patterns of facet misclassification across dimensions?

These questions are intentionally descriptive and exploratory. They are designed to capture patterns of agreement, divergence, and conceptual tension, rather than to test confirmatory hypotheses about the framework’s structure.

### 4.3. Expected Contributions and Scope of Inference

The contribution of this study is methodological and conceptual. First, it provides an empirical documentation of how expert practitioners interpret a fine-grained, multidimensional competency framework at the facet level. Such evidence is rarely reported, despite its importance for the responsible development of complex frameworks.

Second, by identifying facets and dimensions that are consistently interpreted as intended—as well as those that generate ambiguity—the study offers concrete guidance for refining labels, definitions, and explanatory materials. These refinements are particularly relevant for frameworks that are intended to support the instructional design and talent development processes.

Finally, the findings delineate clear boundaries for future research. They inform subsequent stages of validation, including scale development, psychometric modeling, cross-cultural replication, and performance-based assessment. Importantly, the present results should not be interpreted as evidence for the overall validity, reliability, or predictive utility of the ATHENA framework, but as an initial step toward such investigations.

## 5. Method

### 5.1. Participants

The study involved a sample of 75 professionals, comprising 46 instructional designers and 29 human resource development (HRD) professionals. Participants were recruited through professional networks and ongoing organizational collaborations, using a convenience sampling strategy. All participants reported regular involvement in the design, interpretation, or implementation of competency frameworks within educational or organizational settings.

Participants represented a variety of professional sectors, including the banking, construction, retail, and service industries, all located in France. Reported professional experience ranged from 1 to 37 years (M = 11.9, SD = 10.8), and 37.3% of participants held managerial or coordination roles at the time of the study. Gender information was not collected, in accordance with internal data-protection policies in several participating organizations. Age was collected via age categories, ranging from 26 to 30 years old (n = 4), 31 to 40 (n = 10), 41 to 50 (n = 31), and above 50 (n = 30).

Although the sample size and composition do not support generalization beyond this professional context, the participants’ expertise and familiarity with competency-based approaches make them a relevant population for an exploratory evaluation of semantic clarity and interpretability.

### 5.2. Materials

Participants completed an online questionnaire designed to evaluate the semantic clarity and interpretability of the 60 facets of the ATHENA competency framework. For each facet, participants were presented with the following:

- A standardized facet label.
- A concise definition.
- A short contextual or behavioral description, intended to illustrate the construct’s applied meaning.

All materials were presented in French, which corresponds to the participants’ working language. The definitions used in the questionnaire were derived from an iterative process of conceptual synthesis grounded in established psychological theories, and were intentionally formulated as operational hypotheses to be subjected to expert-based evaluation, rather than as finalized theoretical constructs.

No alternative wording or competing definitions were provided, as the aim was to assess the interpretability of the framework as currently specified, rather than to compare alternative formulations.

For each facet, participants responded to two Likert-type items (1 = strongly disagree, 5 = strongly agree):

1 To what extent the definition of the facet is appropriate.
2 To what extent the definition is close to what the participant expected.

In addition, participants were asked to assign each facet to one of the five ATHENA dimensions (cognition, conation, knowledge, emotion, or sensori-motion). Facets were presented in a randomized order, rather than being grouped by dimension, to reduce anchoring effects and encourage independent judgments. Facets were codified for the randomization process, as shown in Appendix B.

### 5.3. Procedure

Participants completed the questionnaire online on a voluntary basis. Before beginning the survey, they received information regarding the purpose of the study, the anonymous nature of data collection, and their right to withdraw at any time. Completion of the questionnaire took approximately 60 min.

The study was conducted as part of a broader human resource management initiative, but the data analyzed here were collected solely for research purposes. The study followed standard ethical principles that were in line with the Helsinki Declaration and complied with European data protection regulations. No personally identifiable information was collected.

### 5.4. Data Analysis

Given the exploratory and pre-validation nature of the study, analyses focused on descriptive and comparative indicators, rather than on inferential modeling of latent structures. For each facet, mean ratings and standard deviations were computed for definition appropriateness and expectation alignment.

To examine the potential differences between instructional designers and HRD professionals, group comparisons were conducted using appropriate non-parametric statistical tests (due to deviations from normal distributions), with corrections applied to control for multiple comparisons. These analyses were intended to detect large or systematic divergences between professional groups, rather than subtle effects.

For the dimensional assignment task, participant classifications were aggregated and compared with the theoretical dimension specified in the ATHENA framework. Facets were considered “misclassified” when the modal dimension selected by participants differed from the theoretical assignment. This analysis was used descriptively to identify patterns of conceptual convergence and divergence across dimensions, not to infer underlying factor structure.

No factor analysis, reliability coefficients, or item-response modeling was conducted, as the study design and measurement instruments were not intended for psychometric validation. All analyses should therefore be interpreted as exploratory and indicative, rather than confirmatory.

## 6. Results

The results are presented in relation to the three research questions, focusing on descriptive patterns of expert judgments regarding facet clarity, expectation alignment, and dimensional interpretability. Given the exploratory nature of the study, the analyses aim to document tendencies and areas of convergence or divergence, rather than to test confirmatory hypotheses.

### 6.1. Appropriateness of Facet Definitions

**Figure 1:**
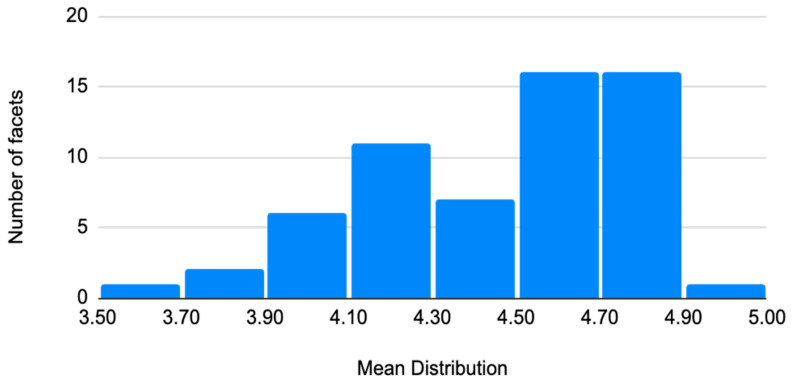
Mean distribution of appropriateness of facet definitions.

In response to the question, “to what extent is the definition of the facet appropriate?”, mean ratings on the 5-point scale were generally high across the 60 ATHENA facets. As shown in Table A1 (Appendix C) and shown in Figure 1 below, mean scores ranged from 3.66 to 4.96, with the median at 4.55 and standard deviations ranging from 0.20 to 1.28, median SD = 0.76. This pattern indicates that respondents tended to agree that the proposed definitions were appropriate for the constructs they were intended to capture.

Only a small number of facets received mean ratings below 4.0, and even in these cases, the values remained above the midpoint of the scale. Overall, the results suggest that, from the perspective of instructional designers and HRD professionals, the ATHENA facet definitions are judged as meaningful and acceptable representations of relevant competencies. Group comparisons between instructional designers and HRD professionals showed that there were no statistically significant differences with the correction for multiple tests in appropriateness ratings, except for two facets—inductive reasoning (U = 381, *p* = .006, r = 0.36) and optimism (U = 390, *p* = .003, r = 0.29)—with instructional designers seeing the definition as less clear than HRD professionals. Based on manager status (manager/non-manager), there were no significant differences except for the inductive reasoning facet (U = 381, *p* = .006, r = 0.36), with managers considering the definition (mean = 4.59, SD = 0.84) more appropriate than non-managers (mean = 4.07, SD = 0.93). There were no differences related to age group, organizational affiliation, or years of experience in the workplace.

### 6.2. Alignment with Participants’ Expectations

**Figure 2:**
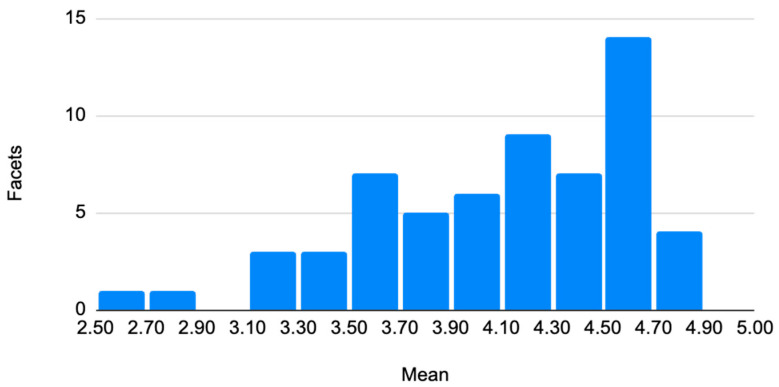
Mean distribution of alignment with participants’ expectations.

The second evaluation question asked: “To what extent is the definition provided close to what the participant expected?” As reported in Table A2 (Appendix D) and shown in Figure 2 below, the mean ratings were again relatively high, ranging from 2.53 to 4.82 (median = 4.25) (SD ranged from 0.39 to 1.53, median = 0.99). For most facets, average scores were close to 4 on the 5-point scale, with standard deviations around 1, indicating that, in general, the formal definitions aligned well with respondents’ pre-existing conceptions of the corresponding competencies. No significant differences emerged between instructional designers and HRD professionals regarding the proximity between definitions and expectations, except for the “heuristics” facet, with pedagogical designers seeing the definition as corresponding to their expectation less than HRD (U = 420, *p* = .009, r = 0.28). There were no differences related to age, manager status, or organizational membership. Concerning years of experience in the workplace, three facets showed significant relationships, with more years of experience being related to seeing that the definitions are more aligned with expectations, (facet 6; r_sp_ = 0.31, *p* = .008, facet 15: r_sp_ = 0.40, *p* = .001; facet 16: r_sp_ = 0.31, *p* = .008).

Only two facets obtained a mean rating clearly below three: facet 45 (“heuristics” 2.53, SD = 1.48) and facet 60, “functional synesthesia” (M = 2.86, SD = 1.42). Heuristics was defined as the capacity to use simplified cognitive strategies to make decisions quickly in uncertain or complex situations, adapting rules to particular contexts (Gigerenzer & Gaissmaier, 2011). Functional synesthesia was defined as the capacity to integrate information coming from different sensory modalities to guide complex motor activity and to handle the variability of sensory stimuli in diverse environments (Stein & Meredith, 1993). The lower rating for expectation alignment suggests that, although participants often judged the definition as meaningful in itself, it did not correspond closely to what they initially imagined under that label. This indicates a potential gap between the theoretical intention of the facet and practitioners’ semantic associations, which may warrant reconsidering either the label, the wording of the definition, or both.

### 6.3. Correspondence Between Theoretical and Perceived Dimension Assignment

Beyond the clarity and expectation alignment of individual definitions, an important question concerns how practitioners locate each facet within the five-dimensional structure of the ATHENA framework. To examine this, participants were asked to assign each facet to one of the five dimensions (cognition, conation, knowledge, emotion, or sensori-motion). Their choices were then compared with the theoretical dimension associated with each facet in the ATHENA framework.

For the majority of facets, the dimension chosen by participants matched the theoretical assignment. However, 13 facets showed a mismatch between the expected dimension and the dominant dimension selected by respondents (see Table 1). This corresponds to 22% of the total set of facets. In several cases, facets that were theoretically assigned to conation or knowledge were instead placed in the cognitive dimension by participants. One sensori-motor facet was also reassigned to conation, and one knowledge facet was frequently located in the emotional dimension. There were no significant differences related to job expertise (instructional designer or HRD), age, managerial status, or organization. These misalignments indicate that, although the facet definitions are generally judged as clear and appropriate, their dimensional placement within the ATHENA architecture is not always transparent to practitioners. The pattern suggests that certain constructs may be conceptually “pulled” toward cognition or other dimensions in applied interpretations, which has direct implications for how the framework is communicated and used in practice.

These results can lead us to reconsider the current configuration of several facets. However, misalignment at the level of expert judgments does not automatically imply that a facet “truly” belongs to another dimension; it may also reflect dominant professional representations (for instance, a tendency to cognitively frame any complex regulation or strategy-like construct).

## 7. Discussion

The present study examined how expert practitioners interpret the facets of a multidimensional competency framework. By focusing on semantic clarity, expectation alignment, and dimensional interpretability, the study deliberately addressed a preliminary but often overlooked stage in the validation of complex frameworks. The results provide a nuanced picture, revealing both substantial areas of conceptual robustness and specific zones of semantic and structural tension.

### 7.1. Semantic Clarity as a Prerequisite Rather than Evidence of Validity

Overall, practitioners judged most ATHENA facet definitions as being appropriate and meaningful. This finding suggests that the framework’s conceptual units are, in general, interpretable by professionals who routinely operationalize competencies in applied contexts. From a validity perspective, this result constitutes evidence related to content- and face-validity at the level of individual facets.

It is essential to emphasize that semantic clarity contributes to psychometric validity. High appropriateness ratings indicate that definitions are intelligible and plausible, but they do not provide information about reliability, internal structure, or predictive utility. In this sense, the present findings should be understood as a necessary but insufficient condition for further validation. Their primary contribution lies in demonstrating that ATHENA’s facets can function as communicable conceptual units, rather than as empirically validated measures.

### 7.2. Expectation Mismatches as Indicators of Conceptual Friction

Ratings of expectation alignment revealed greater variability, with two facets—heuristics and functional synesthesia—standing out as systematic outliers. These mismatches do not necessarily imply that the definitions are incorrect or theoretically unjustified. Rather, they suggest that certain labels evoke professional representations that differ from the framework’s intended meaning.

Such discrepancies are informative. They highlight points where theoretical constructs may be too specialized, insufficiently anchored in practitioners’ everyday language, or conflated with adjacent concepts. From a framework-development standpoint, these results point to concrete candidates for refinement, whether through relabeling, redefinition, or additional explanatory guidance. Importantly, identifying such points at an early stage can prevent downstream measurement artifacts and misinterpretations in later validation phases.

### 7.3. Dimensional Misclassification and the Challenge of Multidimensional Architectures

Approximately one quarter of the facets were systematically assigned to a different dimension than the one specified by the framework. This result directly addresses one of the central challenges of multidimensional competency models: maintaining clear conceptual boundaries between interdependent domains (Katsumi & Dolcos, 2023).

Several patterns emerged. Facets theoretically located in conation or knowledge were frequently reassigned to cognition, reflecting the strong cognitive bias that characterizes many professional representations of competence. Similarly, some sensori-motor facets were interpreted through cognitive or motivational lenses. These tendencies may reflect deeply ingrained disciplinary habits, rather than flaws in the framework itself.

Crucially, dimensional misclassification should not be interpreted as falsification of the ATHENA architecture. Expert judgments reflect dominant interpretative frames, not a latent psychological structure. Nonetheless, these patterns signal zones where the framework’s theoretical distinctions are not immediately transparent to users. For a framework intended for applied use—particularly as an ontology underlying AI-supported systems—such opacity constitutes a practical risk. Clarifying why certain facets are positioned outside the cognitive domain, and making the rationale for these distinctions more explicit, appears to be essential.

### 7.4. Implications for Framework Refinement and Future Validation

Taken together, the findings suggest that ATHENA’s facets form a largely interpretable set of conceptual building blocks, while also revealing specific areas requiring refinement. These insights have direct implications for subsequent research and development.

At the conceptual level, facets that generate systematic ambiguity warrant closer theoretical scrutiny. Some may represent genuinely hybrid constructs, challenging strict dimensional separation. Others may require clearer articulation of their defining features or boundary conditions. At the methodological level, the results inform the design of future validation studies, indicating where item wording, dimensional hypotheses, or measurement strategies should be adjusted before engaging in psychometric modeling.

More broadly, the study illustrates the value of expert-based semantic evaluation as a distinct stage in framework validation. By explicitly examining how practitioners interpret constructs before attempting to measure them quantitatively, researchers can reduce the risk of building psychometric models on semantically unstable foundations.

### 7.5. Limitations

Several limitations must be acknowledged. The study relied exclusively on subjective expert judgments and did not include behavioral tasks, performance indicators, or triangulation with qualitative methods. The sample was limited to French instructional designers and HRD professionals, which restricts generalizability across cultures and professional contexts. In addition, the analyses were descriptive and exploratory, and the results should not be interpreted as evidence for the internal structure or predictive validity of the ATHENA framework (Almanasreh et al., 2019).

These limitations are not incidental; they reflect deliberate methodological choices aligned with the study’s exploratory purpose. Nonetheless, they delimit the scope of inference and underscore the need for subsequent validation work.

The present study does not validate ATHENA in a psychometric sense. Instead, it documents how expert practitioners interpret its facets and dimensional structure, identifying both strengths and points of friction. In doing so, it provides a transparent account of the framework’s current conceptual robustness and its areas of vulnerability.

## 8. Conclusions

This study examined the semantic clarity and interpretability of the 60 facets of the ATHENA competency framework through an expert-based evaluation conducted with instructional designers and human resource development professionals. By focusing on how practitioners understand, differentiate, and categorize the framework’s conceptual units, the study deliberately addressed a preliminary stage in framework validation that is often implicit or overlooked.

The results indicate that most ATHENA facets are perceived as semantically clear and meaningful, suggesting that the framework’s basic conceptual units are broadly interpretable for applied use. At the same time, systematic expectation mismatches and dimensional misclassifications reveal specific zones of conceptual ambiguity. These findings do not undermine the framework’s theoretical ambition but instead provide concrete, empirically grounded signals regarding where conceptual boundaries are less intuitive and where refinement is warranted.

Importantly, the present work does not constitute a psychometric validation of ATHENA. No claims are made regarding its internal structure, reliability, or predictive validity. Rather, the study contributes evidence related to content-level interpretability and expert alignment, clarifying what can—and cannot—be inferred from practitioner judgments at this stage.

By making semantic clarity and conceptual distinctiveness explicit empirical questions, this article contributes to a more rigorous and transparent approach to the development of multidimensional competency frameworks. It highlights the value of expert-based semantic evaluation as a necessary precursor to subsequent psychometric and performance-based validation efforts, particularly for frameworks intended to inform instructional design, talent development, or computational applications.

In this sense, the study should be read not as a conclusion about ATHENA’s validity, but as an informed starting point: one that delineates a clear roadmap for future research while ensuring that subsequent validation efforts rest on conceptually stable foundations.

## Figures and Tables

**Table 1:** Facets misaligned between theoretical assignment and participants’ (experts) assignment.

ATHENA Facet	Framework Dimension	Perceived Dimension
Decision making	conation	cognition
Self regulation	conation	cognition
Perspective taking	emotion	cognition
Post-traumatic growth	emotion	conation
Problem solving	conation	cognition
Continuous learning	conation	knowledge
Cognitive self awareness	knowledge	cognition
Learning strategies	knowledge	cognition
Automation	sensorimotor	cognition
Systemic understanding	knowledge	cognition
Regulatory strategies	knowledge	emotion
Heuristics	knowledge	cognition
Endurance	sensorimotor	conation

## Data Availability

The original contributions presented in this study are included in the article. Further inquiries can be directed to the corresponding author.

## Abbreviations

The following abbreviations are used in this manuscript:

ATHENA	Advanced Tool for Holistic Evaluation and Nurturing of Abilities
HRD	Human resource development

## Appendix A. ATHENA’s 60 Facets Framework and Their Description

ATHENA framework is composed of five dimensions, 19 sub-dimensions, and 60 facets.

(Note that the English translation of definitions and facet names is presented. The original French version is available upon request from the authors).

DIMENSION 1: COGNITION

Reasoning

- Inductive reasoning—Ability to infer general conclusions from specific observations while taking into account contextual particularities.
- Deductive reasoning—Ability to derive specific conclusions from general premises using formal logic, adapting the reasoning process to contextual constraints and specifics.
- Abstract reasoning—Ability to manipulate non-concrete ideas and concepts, identify patterns, and solve problems conceptually, adapting to the complexity and changing nature of environments.

Memory

- Short-term memory—Ability to temporarily retain a limited amount of information for a short period of time, adapting to the specific demands of the task and environment.
- Long-term memory—Ability to store and retrieve information over long periods, organizing and adapting it according to learning and application contexts.
- Working memory—Ability to maintain and structure all information that is necessary for performing complex tasks, adapting to the complexity of professional and organizational demands.

Attention

- Sustained attention—Ability to maintain cognitive focus on a task or specific stimulus over an extended period, adapting to environmental variations and changing task demands.
- Selective attention—Ability to focus on relevant stimuli while ignoring distractions, adapting to the complexity and informational richness of different environments.
- Divided attention—Ability to divide attentional resources across multiple tasks or information sources simultaneously, considering the multitasking demands of various professional contexts.
- Attentional flexibility—Ability to switch efficiently between different tasks or attentional focuses, adapting rapidly to changes in priorities or contexts.

Creativity and Innovation

- Divergent thinking—Ability to generate multiple ideas or original solutions from a single starting point, drawing inspiration from and adapting to various contexts and influences.
- Cognitive flexibility—Ability to adapt one’s thinking and behavior in response to changes in the environment or task demands, integrating new information and perspectives.
- Conceptual synthesis—Ability to combine ideas, information, or existing concepts to create new ideas or solutions, integrating elements from various domains and contexts.

DIMENSION 2: CONATION

Motivation

- Intrinsic motivation—Ability to engage in an activity for the inherent satisfaction it provides, maintaining this engagement despite contextual variations.
- Extrinsic motivation—Ability to engage in an activity to obtain an outcome that is separate from the activity itself, adjusting this engagement based on rewards and consequences that are specific to the context.
- Self-efficacy—Belief in one’s ability to perform the behaviors required to produce specific outcomes, adapting to the complexity and unique challenges of each situation.

Volition

- Self-regulation—Ability to control, monitor, and adjust one’s own behaviors, thoughts, and emotions in order to achieve goals, while adapting to changing environmental demands.
- Perseverance—Ability to maintain effort and commitment toward a goal despite obstacles, adversity, or discouragement, adapting to the nature and intensity of the challenges that are specific to each context.
- Time management—Ability to plan, prioritize, and allocate one’s time efficiently to meet goals, adapting to time constraints and priority demands that are unique to each context.

Proactivity

- Initiative—Ability to act autonomously and proactively without waiting for instructions or external stimuli, identifying and seizing opportunities that are specific to each context.
- Decision-making—Ability to choose or arbitrate between multiple alternatives while considering the potential consequences, using heuristics, strategies, or opportunities that are specific to the context.
- Problem-solving—Ability to identify, define, and solve problems systematically and creatively, adapting to the nature and complexity of the challenges that are specific to each context.

Adaptability

- Behavioral flexibility—Ability to modify one’s behaviors in response to changes in the environment or task requirements, adjusting effectively to new, conflicting, or unexpected situations.
- Uncertainty management—Ability to function effectively in ambiguous or unpredictable situations, developing strategies to cope with the unknown and with change.
- Continuous learning—Ability to actively seek new knowledge and experiences to improve one’s skills, adapting to evolving or changing environmental demands.

DIMENSION 3: KNOWLEDGE

Declarative Knowledge

- Facts—Ability to master an extensive and organized set of precise, objective, and verifiable information, supporting a fine-grained understanding of situations and helping to select the most relevant data for action or decision-making.
- Concepts—Ability to master coherent, structured, and generalizable conceptual representations, facilitating the identification of similarities and differences between categories of ideas, objects, or situations, in order to activate these concepts effectively to clarify, analyze, and structure encountered contexts.
- Principles—Ability to master a structured set of essential rules, norms, or laws that provide a stable and reliable reference framework to guide action, regulate behavior, and orient the interpretation of phenomena according to the constraints and demands of each situation.

Procedural Knowledge

- Technical proficiency- Ability to perform technical tasks effectively and accurately, adapting these skills to the tools, systems, and process requirements that are specific to the context.
- Heuristics—Ability to use simplified cognitive strategies to make quick decisions in uncertain or complex situations, adapting these empirical rules to the specificities of each context.

Metacognitive Knowledge

- Learning strategies—Ability to identify, select, and use effective techniques or methods to enhance one’s learning, adapting these strategies to domain-specific and context-specific learning demands.
- Regulatory strategies—Ability to control, monitor, and adjust one’s behaviors, thoughts, and emotions to achieve goals, adapting to changing environmental demands.
- Cognitive self-awareness—Ability to understand and evaluate one’s own thinking and learning processes, recognizing how they are influenced by different contexts and situations.

Contextual Knowledge

- Knowledge transfer—Ability to apply knowledge and skills acquired in one context to new situations or domains by identifying relevant similarities and differences between contexts.
- Systemic understanding—Ability to perceive and understand complex relationships between the elements of a system or environment, recognizing how these relationships may vary across contexts.
- Cultural intelligence—Ability to understand, adapt, and operate effectively across different cultural contexts, recognizing and respecting culture-specific norms, values, and practices.
- Trend awareness—Ability to identify and anticipate evolutions and changes within a given domain or environment, understanding how these trends may vary depending on context.

DIMENSION 4: EMOTION

Emotional Competence

- Emotional perception—Ability to accurately identify and interpret emotions in oneself and others, considering contextual and cultural cues that influence emotional expression and interpretation.
- Emotional regulation—Ability to modulate one’s emotional states adaptively according to context and goals, taking into account social and professional expectations that are specific to each situation.
- Adaptive use of emotions—Ability to integrate emotional information to guide thinking, decision-making, and behavior constructively, considering the relevance and impact of emotions across contexts.

Resilience

- Stress adaptation—Ability to cope with and positively adjust to stressful or adverse situations by developing and using adaptive strategies that are suited to the specific stress context.
- Optimism—Ability to maintain a positive outlook on future events and see opportunities within challenges, while remaining realistic and adapting to the specific constraints of each situation.
- Post-traumatic growth—Ability to derive positive insights and develop further after difficult or traumatic experiences, integrating this learning in ways that fit different life and work contexts.

Empathy

- Emotional recognition—Ability to identify and understand others’ emotions through verbal and non-verbal signals, taking into account individual, cultural, and contextual differences in emotional expression.
- Perspective-taking—Ability to adopt another person’s psychological point of view, considering their context, experiences, and values.
- Compassion—Ability to feel concern for the suffering of others, coupled with a desire to help them, taking into account cultural norms and contextual constraints that shape the expression and reception of compassion.
- Social intelligence—Ability to understand and navigate effectively within complex social situations, adapting to norms, dynamics, and expectations that are specific to each social context.

DIMENSION 5: SENSORI-MOTION

Perception

- Visual acuity—Ability to discern fine details and process visual information accurately, adapting to lighting, distance, and visual complexity conditions that are specific to each environment.
- Auditory acuity—Ability to detect, discriminate, and locate sounds with precision, adapting to varied acoustic conditions and the auditory demands of different environments.
- Proprioception—Ability to perceive the position, movement, and force of body parts without a visual reference, adapting to various postures, movements, and loads across tasks and environments.

Coordination

- Hand–eye coordination—Ability to synchronize hand movements with incoming visual information, adapting to the precision, speed, and complexity required for various tasks and environments.
- Balance—Ability to maintain a stable posture and control the body’s center of gravity across different positions and movements, adapting to surfaces, disturbances, and equilibrium requirements that are specific to each environment.
- Fine dexterity—Ability to perform precise and controlled movements with hands and fingers, adapting to the size, shape, and texture of manipulated objects and to the precision requirements of each task.

Motor Performance

- Speed—Ability to efficiently execute one or several action sequences rapidly, adapting to the temporal constraints and speed requirements that are specific to each task or environment.
- Precision—Ability to execute movements with accuracy, consistently hitting the intended target or objective, adapting to varying precision requirements across tasks and contexts.
- Endurance—Ability to sustain physical or motor performance over prolonged periods, adapting to different durations, intensities, and types of effort required across tasks and environments.
- Automation—Ability to perform complex motor sequences with minimal conscious cognitive effort, adapting to the variability and complexity of repetitive tasks.

Sensorimotor integration.

- Functional synesthesia—Ability to integrate information from different sensory modalities to guide motor action, adapting to the complexity and variability of sensory stimuli in different environments.
- Sensorimotor adaptation—Ability to rapidly adjust motor responses in reaction to changes in the sensory environment, adapting to variability and unpredictability across contexts.
- Sensory compensation—Ability to use alternative sensory modalities effectively when the usual sensory modality is limited or disrupted, adapting to sensory limitations or disturbances across different environments.

## Appendix B. Naming of the Facets in Table A1 and Table A2

(Note: The original French version is available upon request from the authors).

f1	Inductive Reasoning
f2	Divided Attention
f3	Self-Efficacy
f4	Decision-Making
f5	Concepts
f6	Regulatory Strategies
f7	Cultural Intelligence
f8	Stress Adaptation
f9	Perspective-Taking
f10	Proprioception
f11	Short-Term Memory
f12	Divergent Thinking
f13	Perseverance
f14	Behavioral Flexibility
f15	Technical Skills
f16	Emotional Regulation
f17	Post-Traumatic Growth
f18	Social Intelligence
f19	Fine Dexterity
f20	Sensorimotor Adaptation
f21	Long-Term Memory
f22	Cognitive Flexibility
f23	Intrinsic Motivation
f24	Problem-Solving
f25	Continuous Learning
f26	Cognitive Self-Awareness
f27	Emotional Perception
f28	Emotional Recognition
f29	Speed
f30	Automation
f31	Deductive Reasoning
f32	Working Memory
f33	Selective Attention
f34	Conceptual Synthesis
f35	Extrinsic Motivation
f36	Time Management
f37	Learning Strategies
f38	Systemic Understanding
f39	Auditory Acuity
f40	Precision
f41	Abstract Reasoning
f42	Attentional Flexibility
f43	Self-Regulation
f44	Facts
f45	Heuristics
f46	Knowledge Transfer
f47	Optimism
f48	Hand–Eye Coordination
f49	Endurance
f50	Sensory Compensation
f51	Sustained Attention
f52	Initiative
f53	Uncertainty Management
f54	Principles
f55	Trend Awareness
f56	Adaptive Use of Emotions
f57	Compassion
f58	Visual Acuity
f59	Balance
f60	Functional Synesthesia

## Appendix C

**Table A1:** Descriptive statistics for mean ratings (and SD) of meaningfulness of facet definitions provided.

	N	Mean	Median	SD	Minimum	Maximum
f1	71	4.27	5	0.925	1	5
f2	71	4.30	5	0.932	1	5
f3	71	3.87	4	1.253	1	5
f4	71	4.86	5	0.424	3	5
f5	71	4.07	4	1.046	1	5
f6	71	4.20	4	0.821	1	5
f7	71	4.72	5	0.659	1	5
f8	71	4.77	5	0.484	3	5
f9	71	4.11	4	1.049	1	5
f10	71	4.14	5	1.125	1	5
f11	72	4.64	5	0.698	2	5
f12	72	4.26	5	0.904	2	5
f13	72	4.89	5	0.358	3	5
f14	72	4.76	5	0.489	3	5
f15	72	4.56	5	0.648	3	5
f16	72	4.65	5	0.609	3	5
f17	72	3.96	4	1.204	1	5
f18	72	4.79	5	0.473	3	5
f19	72	4.74	5	0.531	3	5
f20	72	4.18	4	0.877	1	5
f21	68	4.76	5	0.522	2	5
f22	68	4.59	5	0.604	3	5
f23	68	4.43	5	0.739	1	5
f24	68	4.81	5	0.432	3	5
f25	68	4.72	5	0.569	2	5
f26	68	4.43	5	0.759	1	5
f27	68	4.53	5	0.722	2	5
f28	68	4.24	5	1.053	1	5
f29	68	4.66	5	0.536	3	5
f30	68	4.60	5	0.672	3	5
f31	71	4.39	5	1.035	1	5
f32	71	4.03	4	1.095	1	5
f33	71	4.55	5	0.789	2	5
f34	71	4.00	4	1.056	1	5
f35	71	4.14	4	0.961	1	5
f36	71	4.96	5	0.203	4	5
f37	71	4.82	5	0.457	3	5
f38	71	4.55	5	0.650	3	5
f39	71	4.58	5	0.805	1	5
f40	71	4.73	5	0.675	1	5
f41	70	4.63	5	0.705	2	5
f42	70	4.57	5	0.672	2	5
f43	70	4.44	5	0.879	1	5
f44	70	3.96	4	1.279	1	5
f45	70	3.66	4	1.261	1	5
f46	70	4.37	5	0.887	1	5
f47	70	4.63	5	0.705	2	5
f48	70	4.64	5	0.817	1	5
f49	70	4.81	5	0.392	4	5
f50	70	4.29	4.5	0.903	1	5
f51	71	4.80	5	0.496	3	5
f52	71	4.79	5	0.411	4	5
f53	71	4.70	5	0.571	2	5
f54	71	3.82	4	1.073	1	5
f55	71	4.54	5	0.808	1	5
f56	71	4.28	4	0.831	2	5
f57	71	4.42	5	0.981	1	5
f58	71	4.59	5	0.821	1	5
f59	71	4.37	5	0.945	1	5
f60	71	3.97	4	1.069	1	5

## Appendix D

**Table A2:** Descriptive statistics of mean ratings (and SD) of the extent to which the provided definition fit with participants’ expectations.

	N	Mean	Median	SD	Minimum	Maximum
f1	71	3.79	4	1.230	1	5
f2	71	4.01	4	1.201	1	5
f3	71	3.17	3	1.331	1	5
f4	71	4.66	5	0.559	3	5
f5	71	3.65	4	1.148	1	5
f6	71	3.54	4	1.132	1	5
f7	71	4.52	5	0.843	1	5
f8	71	4.72	5	0.614	2	5
f9	71	3.62	4	1.324	1	5
f10	71	3.13	3	1.530	1	5
f11	72	4.35	5	0.952	2	5
f12	72	3.68	4	1.185	1	5
f13	72	4.82	5	0.387	4	5
f14	72	4.67	5	0.581	3	5
f15	72	4.35	5	0.808	2	5
f16	72	4.49	5	0.731	2	5
f17	72	3.47	4	1.332	1	5
f18	72	4.64	5	0.678	2	5
f19	72	4.51	5	0.822	1	5
f20	72	3.46	3	1.074	1	5
f21	68	4.65	5	0.641	2	5
f22	68	4.25	4	0.780	2	5
f23	68	3.94	4	1.105	1	5
f24	68	4.65	5	0.567	3	5
f25	68	4.63	5	0.644	2	5
f26	68	3.71	4	1.037	1	5
f27	68	4.26	5	0.924	1	5
f28	68	3.76	4	1.223	1	5
f29	68	4.50	5	0.838	1	5
f30	68	4.28	5	1.034	1	5
f31	71	4.23	5	1.045	1	5
f32	71	3.52	4	1.319	1	5
f33	71	4.25	5	0.982	1	5
f34	71	3.48	4	1.240	1	5
f35	71	3.55	3	1.169	1	5
f36	71	4.82	5	0.487	2	5
f37	71	4.63	5	0.722	1	5
f38	71	4.11	4	1.008	1	5
f39	71	4.35	5	1.110	1	5
f40	71	4.59	5	0.838	1	5
f41	70	4.26	4.5	0.912	1	5
f42	70	4.29	5	0.965	1	5
f43	70	4.10	4	0.950	2	5
f44	70	3.53	4	1.370	1	5
f45	70	2.53	2	1.481	1	5
f46	70	3.79	4	1.295	1	5
f47	70	4.30	5	0.998	1	5
f48	70	4.49	5	0.959	1	5
f49	70	4.70	5	0.521	3	5
f50	70	3.76	4	1.268	1	5
f51	71	4.54	5	0.842	1	5
f52	71	4.63	5	0.591	3	5
f53	71	4.55	5	0.824	1	5
f54	71	3.17	3	1.121	1	5
f55	71	4.10	4	1.097	1	5
f56	71	4.01	4	0.993	2	5
f57	71	4.38	5	0.976	1	5
f58	71	4.32	5	1.066	1	5
f59	71	3.99	4	1.049	1	5
f60	71	2.86	3	1.417	1	5
